# Meetings of Societies

**Published:** 1912-12

**Authors:** 


### MEETINGS OF SOCIETIES.

### Bristol Medico-Chirurgical Society.

*Annual Meeting, October 9th,* 1912.

After a vote of thanks to the retiring President, Mr. C. A. o rton, had been passed, Dr. Walter Swayne took the chair, and gave an introductory address (*see* p. 316). Later a cordial vote of thanks was given to Dr. Swayne for his able and interesting address.

The Honorary Secretary, Dr. J. A. Nixon, read the following annual report :—

During the year three honorary and fifteen new members have been elected. We have to deplore the death of one member, Dr. Ambrose, and fifteen have resigned, leaving a total membership of 167 and four honorary members.

The balance from the previous year was £114 19s. 11d. From members' subscriptions the receipts were £179 11s. The expenses amounted to £219 14s.

The General Account has £77 11s. 2d. to its credit.

The meetings of the past session were interfered with to some extent by the removal of the Medical Library to the Old Blind Asylum Buildings, and the appropriation to other purposes of the room in which the Society used to meet. Now, however, we are comfortably settled.

A new agreement relative to the Medical Library has been made with the University, and the books have been placed in the new rooms allotted to this library.

The Hon. Librarian, Mr. C. K. Rudge, then read the following report :—

The Library of the Bristol Medico-Chirurgical Society now contains 11,974 volumes. During the year 375 volumes have been added to the Library. The Library is in receipt of 190 periodicals. During the year ninety members have availed themselves of the privilege of taking out books. The number of books borrowed is a third less than the number borrowed during the previous year.

The Society still subscribes to Lewis's Medical Library, and 49 books have been received from this source.

At the request of Professor Walker Hall certain books, illustrating the history of medicine and pathology, have been lent to the Pathological Museum, these books being changed every month.

The Bristol Medical Library, to which members of the Society have access, now contains 22,382 volumes and 249 current periodicals, made up in the following manner :—

BOOKS.

| | |
|---|---|
| Medico-Chirurgical Society .. .. | 11,974 |
| University of Bristol .. .. .. | 8,645 |
| Bristol Royal Infirmary .. .. | 1,189 |
| Bristol General Hospital .. .. | 574 |
| Total .. .. | 22,382 |

CURRENT PERIODICALS.

| | |
|---|---|
| Medico-Chirurgical Society .. .. | 190 |
| University of Bristol .. .. .. | 59 |
| Total .. .. | 249 |

Dr. Shingleton Smith announced his intention of resigning the Editorship of the *Bristol Medico-Chirurgical Journal* at the end of the current year. Dr. Michell Clarke and Mr. Munro Smith expressed the great regret felt by the members of the Society on hearing of the above resignation, and showed how much the Society owed to Dr. Shingleton Smith for the untiring zeal with which he had worked to further the interests of the Society and its *Journal* since their formation in 1874 and 1882 respectively.

Dr. J. A. Nixon announced his resignation of the office of Honorary Secretary to the Society.

Mr. A. J. Wright, M.B., B.S. Lond., F.R.C.S., was elected Honorary Secretary in the place of Dr. Nixon.

The following are the officers of the Society for the Session 1912–13: President—Walter C. Swayne, M.D., B.S. Lond., President-Elect—P. Watson-Williams, M.D. Lond. Committee (*ex-officio*)—C. A. Morton, F.R.C.S.; P. Watson-Williams, M.D. Lond. (Editor of *Journal*); C. K. Rudge, M.R.C.S. (Hon.

Librarian); J. A. Nixon, B.A., M.B. Cantab. (Editorial Secretary); A. J. Wright, M.B., B.S. Lond., F.R.C.S. (Hon. Secretary). (*Elected*)—R. Shingleton Smith, M.D. Lond., and J. Michell Clarke, M.A., M.D. Cantab. (Ex-Presidents); G. Parker, M.A., M.D. Cantab.; I. Walker Hall, M.D. Vict.; F. Lace, F.R.C.S.; J. Lacy Firth, M.D., M.S. Lond., F.R.C.S.; J. O. Symes, M.D. Lond.; L. E. V. Every-Clayton, M.D., B.S. Lond., F.R.C.S.

**RECEIPT AND EXPENDITURE ACCOUNT OF THE BRISTOL MEDICO-CHIRURGICAL SOCIETY, SESSION 1911-12.**

| | £ | s. | d. | | £ | s. | d. |
|---|---|---|---|---|---|---|---|
| Balance from 1911 .. | 114 | 19 | 11 | Honorarium and Stamps | 11 | 0 | 0 |
| Members' Subscriptions | 179 | 11 | 0 | Cheque Book .. .. | 0 | 2 | 6 |
| Paid to Bank on Benson's Cheque .. | 0 | 4 | 0 | Due from 1910–11 .. | 0 | 19 | 3 |
| Interest on Deposit Account .. .. .. | 3 | 0 | 3 | Mitchell & Co. .. .. | 0 | 10 | 6 |
| | | | | Telephone Co. .. .. | 7 | 10 | 0 |
| | | | | Stamps and Postage .. | 2 | 0 | 0 |
| | | | | Weeks & Co... .. .. | 0 | 10 | 6 |
| | | | | Library Grant .. .. | 30 | 0 | 0 |
| | | | | Maggs .. .. .. .. .. | 2 | 5 | 6 |
| | | | | F. Rudge .. .. .. | 2 | 10 | 0 |
| | | | | Lantern .. .. .. | 0 | 10 | 6 |
| | | | | Austen (University Marshal) .. .. .. .. | 1 | 1 | 0 |
| | | | | Lantern .. .. .. | 2 | 2 | 0 |
| | | | | Library Grant .. .. | 50 | 0 | 0 |
| | | | | Mundy & Co. .. .. | 0 | 6 | 0 |
| | | | | Postage .. .. .. .. | 1 | 3 | 0 |
| | | | | To *Journal* .. .. .. | 48 | 0 | 0 |
| | | | | Morgan .. .. .. .. | 0 | 5 | 6 |
| | | | | Rent .. .. .. .. .. | 52 | 0 | 0 |
| | | | | Printing .. .. .. | 6 | 7 | 9 |
| | | | | Balance in Bank, Sept. 30th, 1912 .. .. | 77 | 11 | 2 |
| | | | | Due to Bank .. .. | 0 | 10 | 0 |
| | £297 | 15 | 2 | | £297 | 15 | 2 |

*November 13th, 1912.*

Dr. Walter C. Swayne, President, in the Chair.

Mr. H. Elwin Harris read notes, with comments, on a case of **Congenital Stenosis of the Pylorus.** An infant, soon after birth, began to vomit frequently. The motions at this time were green in colour. At the end of two months the weight was six pounds. In the second month treatment for pyloric stenosis had been begun. The stomach had been washed out daily with sodium sulphate solution, etc. The child remained emaciated, but without increase of emaciation during the third month, and to the middle of the fourth month, and then died suddenly. The speaker referred to the fact that the presence of visible peristalsis is a late symptom in the affection. It might be missed altogether unless specially examined for about ten minutes after food had been taken. In the case related peristalsis had only been felt or seen on three or four occasions. A very thickened pylorus was found *post-mortem*. Reasons were given for regarding the case as one of congenital hypertrophy of the pylorus, and not one of mere spasm, or of spasm with secondary hypertrophy, and the case was considered to point to the advisability of operating if appropriate treatment did not result in improvement after a trial of two or three weeks, and before atrophy of the intestinal mucous membrane had set in.—Dr. C. C. LAVINGTON favoured operation for the relief of the affection, and thought pyloroplasty was probably the best operation. The mortality after gastro-enterostomy was greater.—Dr. STACK thought that many very typical cases had recovered under opium and lavage. He believed that many patients with the affection had curious vomiting and dyspepsia, and that the patients grew very slowly until they were five or six years of age, and after that showed no further symptoms. Diagnostic points were (1) the scanty amount of fæces passed from day to day; (2) the incidence of vomiting without ascertainable cause, *e.g.* in a healthy breast-fed child; and (3) the child often seemed happy and bright immediately after a good vomit. Sudden death was well known in starvation. If a child with the affection was going downhill, operation was indicated even before peristalsis or pyloric tumour could be detected.—Dr. F. H. EDGEWORTH said that statistics showed that four out of five cases recovered without operation. Surgical statistics were not so good. He was doubtful about the advisability of operation if equilibrium had been maintained for three weeks. If the diagnosis was certain, and an operation had to be done, then the sooner it was performed the better. He would expect no gain in weight for some months, and would not consider that occurrence an indication for operation. If vomiting came on three or four weeks after birth it was very suggestive of the affection, and he would treat with lavage and opium, though peristalsis and tumour could not be detected.—Dr. CAREY COOMBS had carefully sought for examples of vomiting in breast-fed infants a few weeks after birth in out-patient hospital practice, but had met with very few examples. He thought there was a discrepancy between the number of cases met with in Bristol and the relatively great number said to be seen at Great Ormond Street Hospital.

Dr. MICHELL CLARKE read notes on a case of **Pyocyaneus Septicæmia.** The patient was a boy of fifteen. The illness began about September 7th, and the patient died on October 3rd. Headache, vomiting and drowsiness were the first symptoms, then muscular weakness and slight cough. Drowsiness increased, headache and photophobia became pronounced. After a fortnight the liver and spleen were enlarged and jaundice marked. Fever was high, and there was a cadaveric-like odour about the patient, probably a diagnostic feature in such cases. Signs of bronchitis were present. Lumbar puncture showed moderate pressure, and the fluid removed contained lymphocytes and epithelioid cells. Acute yellow atrophy of the liver was considered as a diagnosis. There was no leucin or tyrosin in the urine, but crystals of calcium carbonate were plentiful. The bacillus pyocyaneus was obtained from the blood by Dr. Scott-Williamson. At the *post-mortem* the right lung was found to be extensively consolidated, with several scattered abscesses at the base.—Dr. SCOTT-WILLIAMSON pointed out that the organism was not an uncommon one as a contamination in laboratory work. It was often found in fæces. He thought probably a mild pleurisy, with partial collapse of the lower lobes of the lungs, paved the way for the formation of small abscesses, and then infection with the bacillus pyocyaneus followed.—Dr. MUNRO said he had half a dozen experiences of pyocyaneus infection, but not as a pure infection. In two of the cases the infection was a complication of bowel ulceration. Infection with this organism perhaps responded better to vaccine therapy than any other.—Dr. NIXON mentioned a case of galactorrhœa, with eczema of the nipple and breast. A pure culture of the pyocyaneus bacillus was obtained from the milk. A vaccine quickly cured both the eczema and the galactorrhœa.—Dr. EDGEWORTH mentioned a case of ulcerative colitis he had had, in which the above-mentioned bacillus was present. The average temperature was 103°, and half a pint of pus was passed daily. The patient recovered with treatment by cyllin. The patient subsequently came into hospital again with similar trouble, and was a second time cured with cyllin.

Dr. J. O. SYMES read notes on the case of a boy who developed symptoms of **Rheumatic Arthritis at the age of 2 years,** and who was admitted to the General Hospital severely crippled two years later. The distinctive and unusual characteristics of the case were signs of great muscular wasting, together with true rheumatic nodules, and the presence in blood, spleen and glands of a diplococcus resembling the diplococcus rheumaticus. There were also signs pointing to a diagnosis of rheumatoid arthritis, such as fixation of the jaw, swelling of the knee-joints, irregular pyrexia, spondylitis, and enlargement of the lymphatic glands and spleen. Dr. Symes regarded the case as one of general rheumatic infection, illustrating the passage from acute rheumatic fever into rheumatoid arthritis (Still's disease).—Dr. CAREY COOMBS

discussed the relationship between cases such as the above and cases of acute rheumatism.—Dr. J. A. NIXON gave reasons for the opinion he had formed that the above case was an example of fibro-myositis.—Dr. DAVID A. ALEXANDER desired to raise the question of the therapeutical value of sulphur in rheumatoid disease, and especially in large doses. A woman had informed him that she had cured herself of an intractable form, a muscular and articular rheumatism, by taking sulphur and gin, and the quantities were remarkable—an ounce of sulphur to a noggin of gin (5 oz.) every other night: indeed, she took rather more, for she bought ¼ lb. of flowers of sulphur every week for seven weeks, taking in all 28 oz. He had that day seen another patient with osteo-arthritis, unrelieved, although every diagnostic and curative measure had been exhausted upon her—baths, thyroid extract, and an autogenous vaccine, which seemed to be indicated. If in this case he decided to try the sulphur, was he to withhold the gin? The waters of Harrogate might owe their potency to the sulphur they contained, because the aerated medium aided its diffusion and absorption; and the gin, a vaso-dilator and diuretic, may have also greatly furthered the influence of the sulphur. The large doses were followed by no ill-effects, not even by diarrhœa. It was an arsenical compound which brought about what betterment there was in Dr. Symes' patient, with arsenic sulphur was not unallied, and he thought that perhaps in an old woman's remedy he had a secret which would defeat the wisdom of the hospitals.—Dr. SYMES, in reply, fully agreed with Dr. Nixon that the most striking feature of the case was the muscular tenderness, wasting, and contraction, and that this was caused by a rheumatic fibro-myositis.

Dr. FORTESCUE-BRICKDALE read a paper on the **Occurrence and Treatment of Gastric Super-acidity.** Having criticised briefly the classification of functional gastric disturbances, he pointed out the great variations in total acidity and hydrochloric acid content in normal persons, and also in those suffering from conditions commonly considered to be associated with hyperchlorhydria. He considered that primary cases of this condition were a real but rapidly-diminishing group, and noted the occurrence of so called larval cases. He then discussed the relation of the hydrochloric acid to the symptom of pain, and criticised the explanations put forward to account for the latter. This was compared with the pain in organig lesions such as ulcer. He then reviewed the dietetic and druc treatment, and discussed the experimental and clinical basis for both.

J. LACY FIRTH.

A. J. WRIGHT, *Hon. Sec.*

